# Curcumin Affects HSP60 Folding Activity and Levels in Neuroblastoma Cells

**DOI:** 10.3390/ijms21020661

**Published:** 2020-01-19

**Authors:** Celeste Caruso Bavisotto, Antonella Marino Gammazza, Filippa Lo Cascio, Emanuele Mocciaro, Alessandra Maria Vitale, Giuseppe Vergilio, Andrea Pace, Francesco Cappello, Claudia Campanella, Antonio Palumbo Piccionello

**Affiliations:** 1 Department of Biomedicine, Neuroscience and Advanced Diagnostics (BIND), Section of Human Anatomy, University of Palermo, 90127 Palermo, Italy; antonella.marino@hotmail.it (A.M.G.); filocasc@utmb.edu (F.L.C.); mocciaroe@gmail.com (E.M.); alessandra.vitale92@gmail.com (A.M.V.); peppe04@tiscali.it (G.V.); francapp@hotmail.com (F.C.); claudiettacam@hotmail.com (C.C.); 2 Euro-Mediterranean Institute of Science and Technology (IEMEST), 90139 Palermo, Italy; 3 Department of Biological, Chemical and Pharmaceutical Sciences and Technologies (STEBICEF), University of Palermo, 90128 Palermo, Italy; andrea.pace@unipa.it (A.P.); antonio.palumbopiccionello@unipa.it (A.P.P.)

**Keywords:** brain tumors, molecular chaperones, heat shock proteins, HSP60, extracellular HSP60, post-translational modifications, protein folding

## Abstract

The fundamental challenge in fighting cancer is the development of protective agents able to interfere with the classical pathways of malignant transformation, such as extracellular matrix remodeling, epithelial–mesenchymal transition and, alteration of protein homeostasis. In the tumors of the brain, proteotoxic stress represents one of the main triggering agents for cell transformation. Curcumin is a natural compound with anti-inflammatory and anti-cancer properties with promising potential for the development of therapeutic drugs for the treatment of cancer as well as neurodegenerative diseases. Among the mediators of cancer development, HSP60 is a key factor for the maintenance of protein homeostasis and cell survival. High HSP60 levels were correlated, in particular, with cancer development and progression, and for this reason, we investigated the ability of curcumin to affect HSP60 expression, localization, and post-translational modifications using a neuroblastoma cell line. We have also looked at the ability of curcumin to interfere with the HSP60/HSP10 folding machinery. The cells were treated with 6, 12.5, and 25 µM of curcumin for 24 h, and the flow cytometry analysis showed that the compound induced apoptosis in a dose-dependent manner with a higher percentage of apoptotic cells at 25 µM. This dose of curcumin-induced a decrease in HSP60 protein levels and an upregulation of HSP60 mRNA expression. Moreover, 25 µM of curcumin reduced HSP60 ubiquitination and nitration, and the chaperonin levels were higher in the culture media compared with the untreated cells. Furthermore, curcumin at the same dose was able to favor HSP60 folding activity. The reduction of HSP60 levels, together with the increase in its folding activity and the secretion in the media led to the supposition that curcumin might interfere with cancer progression with a protective mechanism involving the chaperonin.

## 1. Introduction

Central nervous system (CNS) tumors are a heterogeneous group of neoplasms with poor prognosis and resistance to therapeutics [1]. During malignant transformation, cancer cells with accumulating genetic and epigenetic abnormalities undergo alteration in protein homeostasis and induction of proteotoxic stress, including dysregulation of the protein translation and protein folding machinery [2,3,4]. These pathways include the dysregulation of proteins involved in the chaperoning system (CS), such as Heat Shock Proteins (HSPs), that are strictly involved in tumorigenesis [5,6]. Human brain tumors also express high levels of many chaperone proteins/HSPs [1,7]. Among these proteins, HSP60 may be a means by which tumor cells can support their high-rate protein synthesis. They can exploit this multifaceted protein to escape from the antitumor immune responses [8,9,10]. This chaperonin is described classically as machinery, with the co-chaperone HSP10, for the folding of other proteins, and it is highly expressed in brain tumors [11,12,13,14]. HSP60 plays either pro-apoptotic or pro-survival functions in a tumor-dependent fashion, and recently it was found that one factor contributing to the multifaceted roles of this chaperonin is its regulation via post-translational modifications (PTMs). For example, it has been demonstrated that HSP60 nitration induces the decrease in ATP-hydrolysis activity, inhibiting the capacity of the chaperonin to fold its preferential substrates [15]. Moreover, HSP60 ubiquitination was associated with necrosis in stress-induced monocyte necrosis with an unclear role [16]. The role of HSP60 in tumorigenesis and progression of brain tumors is still poorly understood. The research aimed at the discovery of HSP60 inhibitors may be attractive and can lead to finding anticancer agents’ adjuvants in combination with chemotherapy, thus to reduce both the adverse effects and drug resistance as well as increase the effective targeting of cancerous cells. In this field, curcumin, which is a polyphenolic compound found in turmeric, with anti-inflammatory, antioxidant, and anti-aggregation properties, has been extensively studied for its neuroprotective effects [17,18,19]. Curcumin has numerous molecular targets, and its anti-tumoral mechanisms of action, including cellular proliferation, apoptosis, autophagy, angiogenesis, immune-modulation, invasion, and metastasis, have been widely studied [20]. Nevertheless, little is still known about its role in ameliorating the stress in cancer and its impact on the components of CS, which are the main regulators of cellular stress. On the other hand, the therapeutic benefits of curcumin, which is known to interfere with protein aggregation in neurodegenerative diseases, appear to be multifactorial and associated with the regulation of transcription factors functioning, or linked with the activity of different proteins [17,18,19,20]. Curcumin and several natural derived-products have been shown to regulate many members of HSPs. These compounds show a significant inhibitory activity of HSP60-induced cell proliferation [21,22]. Therefore, these data encourage the idea that the polyphenolic structure of curcumin might be a recognition motif for HSP60′s binding [22]. Here, we reported the study of the effects of curcumin on HSP60 levels, PTMs, and folding activity using a neuroblastoma cell line. The study aimed to better understand the mechanism at the base of the protection from cellular stress driven by curcumin in a model of tumorigenesis in which HSP60 seems to play a pivotal role. The results obtained showed that curcumin affected the HSP60 levels reducing HSP60 nitration and ubiquitination, favoring its folding activity. These findings are promising to give answers to the opened questions: (i) should curcumin be used as adjuvants in the treatment of brain tumors? (ii) Should HSP60 be a target for curcumin or for curcumin derivatives in the future to be used in the struggle against their devastating effects?

## 2. Results

### 2.1. Curcumin Inhibits Cell Proliferation

**Figure 1:**
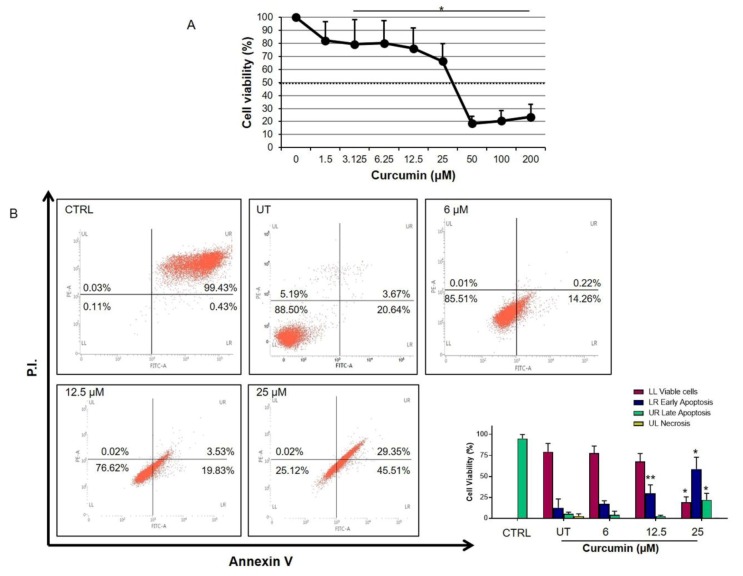
Effects of curcumin on cell proliferation (**A**) MTT (3-(4,5-dimethylthiazol-2-yl)-2,5-diphenyltetrazolium bromide) test analysis. LAN-5 cell viability treated with different curcumin doses (1.5–200 µM) for 24 h using MTT assay. Vertical axis percentage of cell viability; horizontal axis, the concentration of curcumin in micrometers. A dose-dependent decrease in LAN-5 cell viability was observed (growth 50% index = 37.5 μM). * *p* < 0.05 vs. untreated cells (UT). (**B**) Representative flow cytometry graphs are shown for control cells (CTRL, cells treated with dimethyl sulfoxide, DMSO) and for UT and treated with 6, 12.5, and 25 µM of curcumin (P.I.: propidium iodide). The histograms are representative of three independent experiments and show the effect of different doses of curcumin on LAN-5 apoptosis (* *p* < 0.0001 vs. UT; ** *p* = 0.04 vs. UT).

The anti-proliferative effects of curcumin on human neuroblastoma cells (LAN-5) were analyzed by MTT [3-(4,5-dimethylthiazol-2-yl)-2,5-diphenyltetrazolium bromide)] assay (Figure 1A). Cells were treated by increasing concentrations of curcumin (from 0 to 200 µM) for 24 h. A dose-dependent decrease in LAN-5 neuroblastoma cell viability was observed. In particular, the percentage of viable cells decreased significantly when cells were treated with 3.125 µM of curcumin as compared to untreated cells. The growth inhibition index was calculated between 25 and 50 µM (37.5 µM) of curcumin.

Flow cytometry results showed that the percentage of apoptosis of LAN-5 cells treated with curcumin was higher than those of the untreated group in a dose-dependent manner (Figure 1B, *p* < 0.05).

### 2.2. HSP60 Expression after Curcumin Treatments

**Figure 2:**
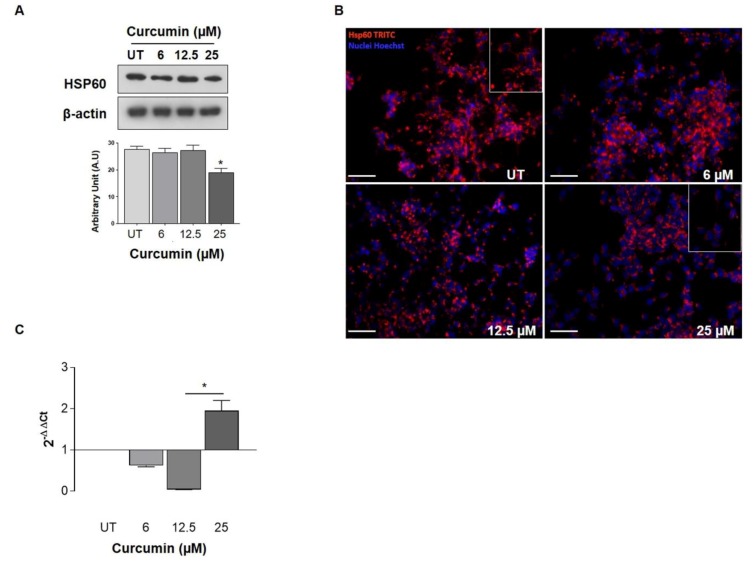
Effect of curcumin treatment on HSP60 expression level (**A**) Representative Western blots and graph of densitometry of the corresponding bands for HSP60 protein expression level in UT, 6 µM, 12.5 µM, and 25 µM of curcumin. β-actin was used as an internal control (* Different than UT, 6 µM, and 12.5 µM, *p* < 0.01). Statistical analysis was performed using ANOVA analysis of variance followed by a Bonferroni post-hoc test. Experiments were performed in quadruplicate. (**B**) Immunofluorescence images confirming the data (Bar: 30 µm). The chaperonin seems to be confined to mitochondria. The insets were obtained using the NIH Image J 1.40 analysis program (National Institutes of Health, Bethesda, MD, USA). (**C**) Representative graph showing real-time PCR analysis of *HSPD1*, (heat shock protein family D, HSP60 member 1) gene expression. The data were normalized to reference genes according to the Livak Method (2^−^^ΔΔCt^). (^*^ Different than UT *p* < 0.05).

**Figure 3:**
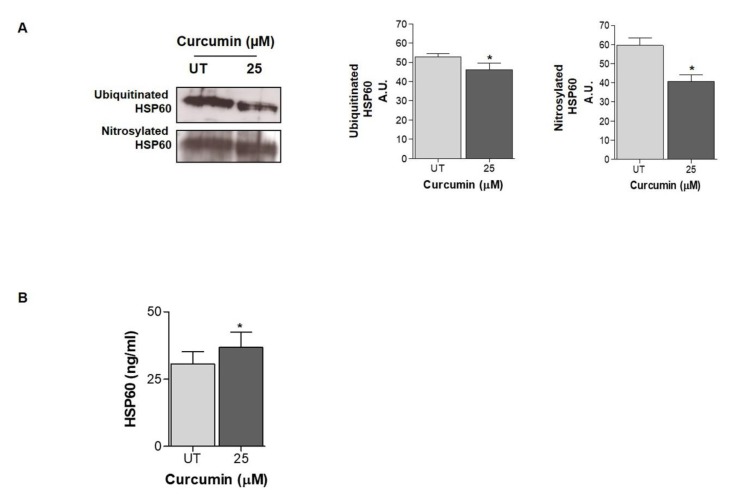
Effects of curcumin treatment on HSP60 post-translational modification and localization (**A**) 25 µM of curcumin does not promote HSP60 ubiquitination and nitrosylation after 24 h of treatment. (**B**) HSP60 levels in cells supernatant, detected by enzyme-linked immunoadsorbent assay (ELISA). The difference between HSP60 levels in cells supernatant after 24 h of treatment (25 µM of curcumin) versus untreated cells was significant (* *p* < 0.001).

Curcumin effect on HSP60 expression was studied. Western blot analysis showed a dose-dependent decrease in HSP60 levels after 24 h of curcumin treatments. In particular, a significant decrease in HSP60 levels was observed after the treatment with 25 µM of curcumin (Figure 2A). These data were confirmed by immunofluorescence. As shown in the inset of the figures, Hsp60 was localized in cellular compartments, resembling mitochondria, and seems not to change this cellular distribution after treatments (Figure 2, inset). Then, it might be reasonable to hypothesize a reduction of the mitochondrial pool protein (Figure 2B). Moreover, HSP60 mRNA expression demonstrated a significant reduction at low concentrations (6 and 12.5 µM) while at 25 µM, HSP60 mRNA levels were increased (Figure 2C). Interestingly, despite the increase in HSP60 mRNA levels observed, there was no increase in HSP60 protein levels. For this reason, we investigated HSP60 PTMs that can be involved in its degradation pathway and cell death. Thus, we first investigated whether curcumin promotes HSP60 ubiquitination, and we observed that ubiquitinated HSP60 levels were lower following treatment with 25 µM of curcumin as compared to untreated cells (Figure 3A; *p* < 0.05). At this point, the fact that HSP60 is not ubiquitinated prompted us to investigate whether curcumin may promote different post-translational changes, recently investigated by our research group in another cancer in vitro model. Since the role of S-nitrosylation has been widely studied in cancer, we evaluated the effects of curcumin on the HSP60 S-nitrosylation level. A significant decrease in the levels of nitrated HSP60 was observed after the incubation with 25 μM of curcumin for 24 h compared to untreated cells (Figure 3A; *p* < 0.05).

### 2.3. HSP60 Is Released in the Extracellular Environment after Curcumin Treatment

Previous observations have shown that HSP60 is released by cancer cells in the extracellular microenvironment, where it can affect the properties of the tumor microenvironment [23,24,25,26]. To further investigate and explain the decreased HPS60 protein levels despite the increase in HSP60 mRNA levels, we detected the HSP60 levels in cell supernatant by an enzyme-linked immunoadsorbent assay (ELISA) assay. The extracellular HSP60 levels were increased when cells were treated with 25 µM of curcumin (Figure 3B).

### 2.4. Curcumin Increases HSP60 Folding Activity

**Figure 4:**
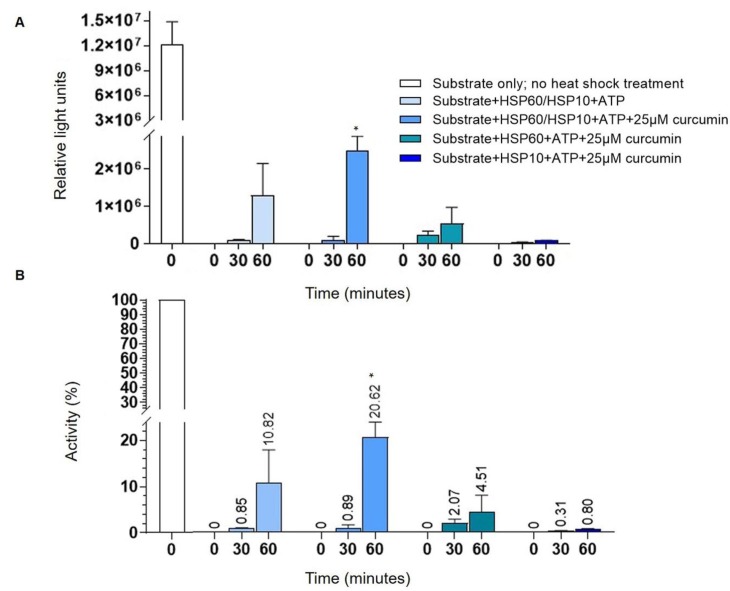
Folding test: At indicated times 30–60 min, aliquots were taken from each of the reactions and added to assay wells containing 50 µL of Luciferin reagent. Luminescence measurements were taken using a GloMax^®^ 96 Microplate Luminometer (Promega Corporation, Madison, WI, USA) (Substrate: Glow-Fold™ Substrate Protein). (**A**) The graph shows the average of three independent experiments. The folding test demonstrated that curcumin (25 µM) significantly promoted the refolding activity of HSP60/HSP10 complex after 60 min of reaction (* *p* < 0.01 vs. Substrate + HSP60/HSP10 + ATP, Substrate + HSP60 + ATP + 25 µM curcumin, Substrate + HSP10 + ATP + 25 µM curcumin, and Substrate only; no heat shock treatment). Furthermore, the test showed that curcumin had no effects on the activity of the complexes, which were not present, respectively, HSP10 and HSP60. After 30 min of reaction, we observed no significant changes in folding activity in the presence of curcumin (Substrate + HSP60/HSP10 + ATP + 25 µM curcumin) and in the absence of HSP10 (Substrate + HSP60 + ATP + 25 µM curcumin) or HSP60 (Substrate + HSP10 + ATP + 25 µM curcumin), when compared with the reaction without curcumin (Substrate + HSP60/HSP10 + ATP, at 30 min). (**B**) The graph shows the percentage of the refolding in all conditions used, compared with the luciferase activity in the presence of the substrate not heated (Substrate only; no heat shock treatment), considered 100% of activity. (* *p* < 0.01 vs. Substrate + HSP60/HSP10 + ATP, Substrate + HSP60 + ATP + 25 µM curcumin, Substrate + HSP10 + ATP + 25 µM curcumin, and Substrate only; no heat shock treatment).

The effects of curcumin on HSP60 folding activity was evaluated by conducting an in vitro assay in the presence or absence of 25 µM of curcumin. Luminescence data were reported as average luminescence (relative light units) (Figure 4A) and as a percentage of the refolding (Figure 4B) measured from three independent experiments. The refolding activity of HSP60 in the presence of 25 µM of curcumin was compared to the control and other conditions (respectively, no heat-shocked substrate; without curcumin; HSP10 Solution; HSP60 Solution). We investigated whether the binding of curcumin to the HSP60/HSP10 complex has any consequences for its folding activity. After 30 min of incubation with Luciferin reagent, we did not observe any significant changes in refolding activity between conditions used. Instead, after 60 min of reaction, the substrate was refolded more effectively in the presence of curcumin (Figure 4). According to the characteristic function of HSP60, its protein-folding process occurs in cooperation with the co-chaperonin HSP10, as demonstrated by the assay. In fact, we observed an inhibition of HSP60 chaperone activity in the absence of HSP10 (Figure 4). These data demonstrated that curcumin increased the folding activity of HSP60/HSP10 complex, most probably stabilizing the complex by interacting with the folding site of the protein and promoting the folding activity (Figure 4). Furthermore, the association with the co-chaperone HSP10 to HSP60 subunits helps promote the refolding of the substrate (Figure 4) [8].

## 3. Discussion

In this study, we investigated the anticancer properties of curcumin in a neuroblastoma cell line with a focus on HSP60. The results show that curcumin is cytotoxic in the human neuroblastoma cell line through the induction of apoptosis. This effect could be associated with the reduction in intracellular HSP60 levels and its release in the extracellular space. Moreover, it is well known that curcumin presents an anti-aggregation effect in a different class of proteins by modulating their conformational stability and unfolding/folding pathways [27]. Concerning the effect on HSP60, we found that curcumin can increase this folding activity, which is central in the control protein homeostasis and the regulation of proteotoxic stress in cancer [28].

Curcumin has been shown to have various properties in human cells, such as antioxidant [29], anti-inflammatory [30], antimicrobial [31], and anticancer properties [32,33,34]. Curcuma longa, the plant from which curcumin is extracted, has been traditionally used in Asian countries as a medical herb for the care of several pathologies and has been widely used to prevent neurodegenerative diseases. Curcumin may be considered a neuroprotective agent because of its ability to modulate many molecular targets, such as transcription factors, inflammatory cytokines, kinases, growth factors, and antioxidant systems. Several studies have highlighted that curcumin appears to be responsible for the improvement of neuroprotective actions via the inhibition of oxidative damage [35] and mediating the reduction of microglial inflammation [36].

The growing interest in therapeutic uses of curcumin on neurological diseases [37] may open new insight on the elucidation of its biological effects on neuronal cell models. Curcumin is known to decrease neuroinflammation and enhance microglial phagocytosis [38]. In neurodegenerative disorders, in which the protein aggregation of misfolded proteins is implicated, curcumin increases the induction of HSPs [39]. Moreover, curcumin can inhibit reactive oxygen species (ROS) formation and trigger mitochondrial protection and anti-apoptotic mechanisms [36,40]. Many investigations have been done in the attempt to use chaperones for the corrections of protein misfolding and treatment of human diseases [41]. Among HSPs, HSP60 has been shown to be involved in brain tumors [1,42,43,44], and preclinical in vitro and in vivo data have shown that curcumin may be an effective treatment for brain tumors [17]. Nevertheless, it remains to be explored what the effect of curcumin on HSP60′s expression and/or function is. Therefore, a detailed investigation of the curcumin-HSP60 relationship on neuronal cells is fundamental for the further development of curcumin-based treatment or perspective curcumin-like drugs.

We started the biochemical characterization by assessing the toxicity profile on our cellular model using the MTT test. Despite the well-known curcumin cytotoxicity [45], cell death was evidenced at relatively high doses (>50 μM), allowing the selection of lower doses as test conditions for subsequent analysis. Our results demonstrate, in agreement with previous results [46,47,48,49], that curcumin induces apoptosis in human neuroblastoma cells in a dose-dependent manner, as detected by Annexin V/PI test. Considering the effect of curcumin on HSP60 expression, the reduction of the expression at higher doses is noteworthy, while the HSP60 mRNA expression decreased after lower doses treatment but consistently increased after 24 h at 25 μM. These divergent data suggest that the low levels of the HSP60 protein in curcumin-treated cells are due to post-transcriptional processes and subsequent HSP60 degradation and/or extracellular releasing and do not involve gene expression downregulation.

We have already observed, in other cancer cell models, the reduction of the HSP60 levels after the treatment with drugs or cytotoxic compounds that can affect directly and/or indirectly the HSP60 functions. In a mucoepidermoid carcinoma cell line (NCI-H292), we demonstrated that the high levels of HSP60 caused the inhibition of the pro-caspase 3 (pC3) activation and the resistance to apoptosis. The treatment with a copper compound with cytotoxic properties induced the decrease in the HSP60 levels, the separation of the complex HSP60/pC3, and consequently, the C3 activation of the caspase pathway associated with a tumor-cell growth arrest [50]. In addition, we found that the antitumor agent doxorubicin acted on the NCI-H292 cell line by lowering the levels of intracellular HSP60 and determining the HSP60 acetylation, which seems to be responsible for the disruption of Hsp60/p53 complex and the activation of replicative senescence, probably via p53-p21 pathway [51]. Our previous findings reported that high levels of HSP60 may have an anti-apoptotic and cytoprotective role in cancer [15,50,51,52]. Compounds that cause the reduction of HSP60 levels may have various effects, as has already been demonstrated. HSP60 can be tagged by PTMs that can regulate its degradation and/or secretion [15,23,50,51,53,54]. Thus, we turned our attention to possible PTMs of HSP60 induced by curcumin. Our results demonstrate that curcumin did not promote HSP60 ubiquitination and, then we explored the S-nitrosylation. In a previous work [15], we showed that the anti-cancer drug Suberoylanilide hydroxamic acid (SAHA), induces HSP60 nitration in mucoepidermoid tumor cells and we postulated that the nitration may occur in highly conserved Tyr222 and Tyr226 in the apical domain of HSP60 affecting the substrate binding and interfering with HSP60 folding activity [15,55]. The S-nitrosylation has been studied mainly in tumor cells [56,57,58] and seems to be a PTM that contributes to cell proliferation and expansion in glioma [59,60]. Nitrosative stress represents a threat to the cell folding machinery, and it has been shown that the S-nitrosylation affects different pathways leading to the inhibition of microglial caspase-3 in glioblastoma and the determination of pro-tumor events activation [60]. Our results demonstrated that curcumin reduces the HSP60 S-nitrosylation, confirming its ability to mitigate the effects of oxidative stress and restore proteins’ functions to levels observed under homeostasis. We also demonstrated that curcumin promotes and improves the folding activity of HSP60. Indeed, curcumin’s effects can also be related to the direct interaction with this chaperone, suggesting a positive effect on the HSP60/HSP10 complex or for client protein ligation. While the possible mode of action of HSP60 inhibitors is well established [61,62,63], the binding site of curcumin for HSP60/HSP10 folding machine is unknown, and, to the best of our knowledge, this is the first case of enhancer of this CS.

At this point, it remains unclear why intracellular HSP60 is low in curcumin-treated cells, and at the same time, HSP60 expression is not downregulated and, as mentioned above, the HSP60 ubiquitination seems to be not involved either. Therefore, to explore potential mechanisms underlying the decrease in the HSP60 intracellular levels in curcumin-treated cells, we evaluated possible extracellular release. Other groups and we have already shown that HSP60 can be secreted by cells, in particular by cancer cells via both secretion canonical pathways and via exosomes [15,23,24,26,50,64,65,66,67]. In this work, we demonstrated that HSP60 is secreted by LAN-5 cells after 24 h of treatment with 25 µM of curcumin. HSP60 has different roles in the extracellular environment in healthy and diseased tissues. HSP60 has been found on the surface in both normal and tumor cells, where may be implicated in transmembrane transport and signaling and the immune system activation [10]. The HSP60 secretion is linked to widespread intercellular communication events with various biologic effects. In brain tumors, HSP60 is overexpressed [68,69], and its secretion may be a mechanism involved in cell transformation and in tumor progression, suggesting that HSP60 is a potential therapeutic target for brain tumors treatment [42].

Considering its anti-oxidant and anti-inflammatory properties, the perspective use of curcumin as adjuvant chemotherapy in brain cancer is promising. In this work, we highlighted the effect of curcumin’s treatment on the apoptosis induction of neuronal cells, showing the possible effect on the expression and activity of HSP60, a member of chaperones family with growing interest particularly for its involvement on tumor progression.

## 4. Materials and Methods

### 4.1. Cell Culture and Treatments

The human neuroblastoma cells (LAN-5), kindly provided by the National Research Council of Italy (CNR, Palermo, Italy), were grown in Roswell Park Memorial Institute (RPMI) 1640 with 10% heat-inactivated fetal calf serum (FCS) and supplemented with 2 mM glutamine, 50 U/mL penicillin, and 50 mg/streptomycin in a humidified incubator containing 5% CO_2_ at 37 °C. The passage number of cells used in this study ranged from 12 to 35. Unless otherwise stated, cell culture reagents were purchased from GIBCO BRL LIFE Technologies (Waltham, MA, USA). Before all the experiments, 8 × 10^3^ cells/well in 96-well-plates and 1 × 10^6^ cells in 25 cm^2^ flasks were seeded, and confluent cell monolayers were incubated in a serum-free medium for 24 h. For MTT cells were seeded into 96-well tissue culture plates, and 8-well chamber slides, respectively. For cytometry analysis, protein and RNA extraction, cells were seeded into 25 cm^2^ flasks, and for immunofluorescence assays, cells were plated onto 8-well microscope chamber slide. One day after seeding, cells were treated for 24 h with different concentrations of curcumin (0–200 µM). Curcumin was dissolved in 75% ethanol to a final concentration of 1 mM. The successive dilutions were carried out using the culture medium.

### 4.2. Cell Proliferation Assay

The cytotoxic effect was determined by MTT cell viability test. MTT was obtained from Sigma (Milan, Italy), and the assay was performed as described [15,50]. Briefly, after 24 h of treatments, the medium containing the compounds was replaced, and MTT was dissolved in fresh medium and added to the cell cultures at a final concentration of 0.5 mg/mL. Following a 4 h incubation period, cells were solubilized in 200 µL DMSO/well, and optical density (OD) was measured with a plate reader (Titertek Multiskan MCC/340, Flow Laboratories, McLean, VA, USA) at 570 nm (630 nm as reference). Cell viability was expressed as the percentage of the OD value of inhibitor-treated cells compared with untreated controls, according to the following equation: Viability = (OD SAMPLE/OD CONTROL) × 100. Each experiment was carried out in duplicate, and three experiments were performed for the compound.

### 4.3. Flow Cytometry

The cells were seeded into 25 cm^2^ flasks at a density of 1 × 10^6^ cells/flask and were treated with different concentrations of curcumin. A positive control group (DMSO treated cells) was set up. After 24 h of treatment, the cells in each group were collected by centrifugation. The apoptosis was measured using an Annexin V-FITC Apoptosis Detection Kit (Abcam, Cambridge UK). Five hundred microliters of 1×binding buffer was used to re-suspend cells, and 5 μL of Annexin V-FITC and 5 μL of PI were added. Blank control and single staining control groups were set up. Apoptosis was detected using a BD FACSVerse™ flow cytometer (BD Biosciences, Franklin Lakes, NJ, USA). The experiment was repeated 3 times.

### 4.4. Western Blotting

Treated and untreated cells were lysed into ice-cold lysis solution containing RIPA buffer, as previously described [15,50]. Lysates were then spun at 16,000× *g* for 30 min at 4 °C, the supernatant was recovered, the protein concentration determined, and then stored at −80 °C until use. Proteins were quantified with the Quant-iT^TM^ protein assay kit (Invitrogen Molecular Probes, Eugene, OR, USA), using the Qubit fluorimeter according to the manufacturer’s instructions (the kit is accurate for protein concentrations ranging from 12.5 mg/mL to 5 mg/mL).

Western blotting analyses of cell lysates were performed as previously described [51]. Briefly, 20 µg of proteins from cell lysates were added to 4×Laemmli buffer and heated for 5 min at 95 °C. Proteins were resolved by 12% SDS-PAGE along with a molecular weight marker (Bio-RAD laboratories, Milan, Italy). Proteins were then transferred to nitrocellulose membranes. After transfer, all membranes were stained with Ponceau S to verify the quality of transfer and loading similarity. Before applying antibodies (Table 1), the membranes were blocked with 5% BSA and probed for 12 h with the specific antibody, followed by incubation with horseradish peroxidase-conjugated second antibody. Blots were detected using the Supersignal West Femto, according to the manufacturer’s instructions (Pierce Thermo Scientific, Waltham, MA, USA), and chemiluminescent signals were recorded with the ChemiDoc XRS imager (Bio-RAD Laboratories, Milan, Italy). Densitometric analysis of blots was performed using the NIH Image J 1.40 analysis program (National Institutes of Health, Bethesda, MD, USA).

### 4.5. Real-Time Quantitative PCR (qPCR)

Total cellular RNA was isolated from cell cultures using TRIzol^®^ REAGENT (Sigma–Aldrich, Milan, Italy), according to the manufacturer’s instructions. RNA (12 ng) was retro-transcribed using the ImProm-II Reverse Transcriptase Kit (Promega Corporation, Madison, WI, USA) to obtain cDNA, which was amplified using the GoTaq qPCR Master Mix (Promega Corporation, Madison, WI, USA) as previously described [70]. The mRNA levels were normalized to the levels obtained for hypoxanthine phosphoribosyltransferase 1 (HPRT1), for beta-glucuronidase (GUSB) and for Glyceraldehyde 3-phosphate dehydrogenase (GAPDH), which primers sequences are indicated in Table 2. Changes in the transcript level were calculated using the 2^−ΔΔCt^ method [71].

### 4.6. Immunofluorescence

Immunofluorescence was performed as described before [52]. Cells were fixed with ice-cold methanol for 30 min, washed in phosphate buffer solution (PBS), pH 7.4, and were incubated with the unmasking solution (trisodium citrate 10 mM, 0.05% Tween 20) for 10 min at room temperature (RT). Then, the cells were incubated with the blocking solution (3% albumin bovine serum in PBS) for 30 min at RT and with HSP60 primary antibodies, overnight at 4 °C. The next day, the cells were incubated with fluorescent secondary antibodies mouse IgG antibody conjugated with Texas Red (Sigma–Aldrich, Milan, Italy) diluted 1:200 for 1 h, at RT. The nuclei were counterstained with Hoechst 33,342 (Sigma–Aldrich, Milan, Italy) for 15 min at RT. Finally, all slides were mounted with coverslips using a drop of PBS, and then imaging was performed using a Leica DM5000 upright fluorescence microscope (Leica Microsystems, Heidelberg, Germany).

### 4.7. Immunoprecipitation Analysis

To detect HSP60 post-translational modifications, immunoprecipitation was performed as previously described [15]. Briefly, 5 μg of anti-HSP60 antibody per 500 µg of total cell lysate was incubated overnight at 4 °C with gentle rotation. Antibody/protein complexes were then immunoprecipitated with antibodies linked to Protein-G/A Sepharose beads. Nonspecifically bound proteins were removed by repeated washings with isotonic lysis buffer. Immunoprecipitated proteins were resolved by 12% SDS-PAGE using primary antibodies against ubiquitin and 3-nitrotyrosine.

### 4.8. Enzyme-Linked Immunoadsorbent Assay Test

Enzyme-linked immunoadsorbent assay (ELISA) was performed as described [71,72] using a commercial human HSP60 ELISA kit (StressMarq, Biosciences Inc., Victoria, BC, Canada) and absorbance was measured at 450 nm in a microplate photometric reader (GDV, Milan, Italy).

### 4.9. Folding Test

The folding test was conducted using the HSP60/HSP10 Glow-Fold^TM^ Protein Refolding kit (Boston Biochem, Cambridge, MA, USA) following the manufacturer’s instructions. Briefly, for the test conditions, we prepared reactions using, 10X Reaction Buffer, 5X HSP60 Solution, 10X HSP10 Solution, 10X Mg^2+^-ATP solution, 10X Glow-Fold™ Substrate Protein, and 25 µM of curcumin. For control conditions, we omitted, respectively, curcumin, HSP10 Solution, HSP60 Solution, and we included a positive control in which the Glow-Fold™ Substrate Protein was not denatured. The Glow-Fold™ Substrate Protein was denatured through heat shock (for 7 min at 45 °C). Refolding reactions were conducted at 30 °C for 30 min and 60 min, and the luminescence was measured by the addition of Luciferin Reagent, using a luminescence capable plate reader GloMax^®^ 96 Microplate Luminometer (Promega Corporation, Madison, WI, USA), within 1 min of mixing. Luminescence data were reported as relative luminescent values (relative light unit) of refolding activity, measured for the positive control (Glow-Fold™. Substrate protein no heat-shock, at time = 0) and for reaction conditions (at time = 30 min and 60 min). In addition, data were reported as the percentage of activity when compared with the luciferase activity in the presence of the substrate not heated (positive control), considered 100% of activity. Experiments were performed in triplicate.

### 4.10. Statistical Analysis

Data are presented as the mean ± S.D. of triplicate determinations. Comparisons between groups were performed using the statistical software package GraphPad Prism^TM^ 4.0 software (GraphPad Prism^TM^ Software Inc, San Diego, CA, USA), and SigmaPlot11 (Systat Software Inc., San Jose, CA, USA) with the unpaired samples Student’s *t*-test and one-way ANOVA analyses. A *p*-value < 0.05 was considered statistically significant.

## 5. Conclusions

In conclusion, HSP60 is a chaperonin with an active role in proliferation and neoplastic transformation. The HSP60 levels are increased in a number of tumors, in which it may act in different manner and may be found not only at intracellular level, but also extracellularly and in circulation [5,6,9,10]. In the present study, we found that curcumin induces the cell death by apoptosis in a neuroblastoma cell line and modify levels and biochemical characteristics of HSP60. In fact, after treatment with 25 µM of curcumin the HSP60 intracellular levels were reduced, whereas the extracellular levels were increased. Furthermore, curcumin is able to increase the refolding activity of the chaperone system HSP60/HSP10. The beneficial effect of curcumin requires further elucidation, and our findings may be helpful in to design anti-cancer therapeutic strategies, using curcumin and exploiting its influence on HSP60.

## Figures and Tables

**Table 1:** Primary antibodies used for Western blot, immunofluorescence, and immunoprecipitation analyses.

Method	Antigen	Type and Source	Clone	Supplier	Catalogue No.	Dilution
WB, IF	HSP60	Mouse monoclonal	LK-1	Sigma–Aldrich	H-4149	1:1000, 1:100
WB	β-actin	Mouse monoclonal	C-4	Santa Cruz Biotechnology	AC-15	1:3000
WB	ubiquitin	Mouse monoclonal	P4D1	Santa Cruz Biotechnology	sc-8017	1:1000
WB	3-nitrotyrosine		39B6	Abcam	ab-61392	1:1000
IP	Protein A PLUS-Agarose	Mouse monoclonal		Santa Cruz Biotechnology	sc-2003	20 µL

Abbreviation: WB, Western blot analysis; IF, immunofluorescence, IP, immunoprecipitation.

**Table 2:** Forward and reverse primers used for quantitative real-time PCR (qRT-PCR).

Primer	Forward	Reverse
GUSB	5’-ACCACCCCTACCACCTATATC-3’	5’-ATCCAGTAGTTCACCAGCCC-3’
GAPDH	5’-GAAACCCATCACCATCTTCC-3’	5’-TCCACGACATACTCAGCAC-3
HPRT1	5’-TGTCATGAAGGAGATGGGAG-3’	5’-ATCCAGCAGGTCAGCAAAG-3’
HSPD1 var1	5’-GAGTAGAGGCGGAGGGAG-3’	5’-AGTGAGATGAGGAGCCAGTA-3’

Abbreviation: GUSB, beta-glucuronidase; GAPDH, glyceraldehyde-3-phosphate dehydrogenase; HPRT1, hypoxanthine phosphoribosyltransferase 1; HSPD1 (heat shock protein family D, Hsp60 member 1).
